# Musculoskeletal Manifestations of Alkaptonuria: A Case-Based Review of Literature

**DOI:** 10.7759/cureus.90841

**Published:** 2025-08-23

**Authors:** Anjali K, Suresh Kumar, Bhargavi M V, Srinivasan Ramadurai, Rajkumar Mani

**Affiliations:** 1 Internal Medicine, Sri Ramachandra Institute of Higher Education and Research, Chennai, IND

**Keywords:** alkapton bodies, alkaptonuria, homogentisate deoxygenase, intervertebral disc calcification, ochronotic arthropathy

## Abstract

Alkaptonuria is a rare metabolic disorder caused by a mutation in the homogentisate 1,2 dioxygenase (HGD) gene, which encodes the HGD enzyme. It is inherited in an autosomal recessive pattern. This leads to the accumulation of homogentisic acid (HGA) and benzoquinone acetic acid (BQA), which is an oxidised product of HGA. Both these compounds form polymerised deposits, which cause bluish black discolouration of the cartilage. They also cause inflammation, degeneration and calcification of ligaments, large joints, tendons and intervertebral discs. A fragmented cartilage forms, which attaches to the synovial membrane and causes subsequent degeneration and fibrosis, leading to ochronotic arthropathy. Both HGA and BQA are deposited in the bone and impair bone mineralisation, which leads to osteopenia and osteoporosis.

We report a case of a 63-year-old gentleman with type 2 diabetes mellitus who presented with a history of chronic backache and acute urinary retention for one day. The patient also gave a history of urine turning dark on standing. Clinical examination revealed blackish sclera, kyphoscoliosis, and deformity of the distal interphalangeal joints of the hand. CT scan of the abdomen revealed multiple calculi within the prostatic urethra and the bulbar urethra, which explained the acute urinary retention. X-ray of the cervical spine showed narrowing of intervertebral spaces and straightening. X-ray of the dorso-lumbar spine showed kyphoscoliosis, calcification of intervertebral discs, subchondral sclerosis and disc space narrowing. X-ray of bilateral knee joints showed multiple osteophytes with joint space reduction and features of severe osteoarthritis of the knee. CT scan of the dorso-lumbar spine showed dorso-lumbar scoliosis of the vertebra, extensive ankylosis, diffuse osteopenia, disc space narrowing, multiple intervertebral disc space calcification and osteophytes involving the entire vertebrae. Urine HGA assay was found to be elevated (1161 mmol/L, normal < 1), which was diagnostic of alkaptonuria. The patient later underwent percutaneous cystolithotripsy. Following the procedure, blackish stones were retrieved, suggestive of alkapton bodies. Given the advanced stage of musculoskeletal manifestations, the prognosis was poor, and the patient was started on physiotherapy along with analgesics to help with mobility.

Early identification of ochronotic arthropathy is imperative to slow the progression of the disease, to reduce morbidity, to improve the quality of life and to facilitate surgical intervention when necessary. We have reviewed the literature to summarise the musculoskeletal manifestations, radiological findings in alkaptonuria and the treatments that are available currently to treat alkaptonuria.

## Introduction

Alkaptonuria is a relatively rare metabolic disorder, inherited in an autosomal recessive pattern. It is caused by a mutation in the homogentisate 1,2 dioxygenase (HGD) gene, which encodes HGD, which is involved in tyrosine metabolism [1-2]. The gene encoding HGD has been localised to human chromosome 3q21-q23 [3]. Gene mutation causes a deficiency of the HGD enzyme. It leads to increased levels of homogentisic acid (HGA), which is eliminated in the urine (homogentisic aciduria) or is deposited after polymerisation in cartilages and bone, leading to arthritis and joint destruction. HGA in urine turns black or dark brown on alkalinization/oxygenation. Major musculoskeletal manifestations of alkaptonuria are ochronosis and spondyloarthropathy involving the axial and appendicular skeleton, with sparing of the sacroiliac joint, with onset between 40 and 50 years of age. Other manifestations of alkaptonuria include renal, prostatic and urethral calculi, as well as cardiovascular abnormalities like coronary artery calcification and aortic stenosis [4].

We report a case of a 63-year-old gentleman who was on treatment for type 2 diabetes mellitus (T2DM) with a history of chronic backache for the past 20 years, who presented to our hospital with acute urinary retention.

## Case presentation

A 63-year-old gentleman with a history of T2DM, on treatment with oral antidiabetic drugs, with no other comorbidities, presented to our hospital with a history of acute urinary retention for one day. His medications included metformin tablet 500 mg twice a day, sitagliptin tablet 100 mg once a day and multivitamins. 

On further probing, the patient gave a history of low backache for the past 20 years, which was progressive and associated with morning stiffness. He also reported knee pain, which had worsened over the last five years, restricting his daily activities. The patient stated that his symptoms started in his early 40s as intermittent lower back pain, which then became persistent over the years, and it was associated with morning stiffness, which lasted for one hour. He noticed decreased flexibility of the spine in the form of difficulty while bending forward. The patient also gave a history of urine turning dark on standing. The patient’s joint symptoms were empirically treated as rheumatoid arthritis for some duration in a local clinic, with no improvement; hence, he stopped seeking medical care for his joint symptoms after that. 

**Figure 1:**
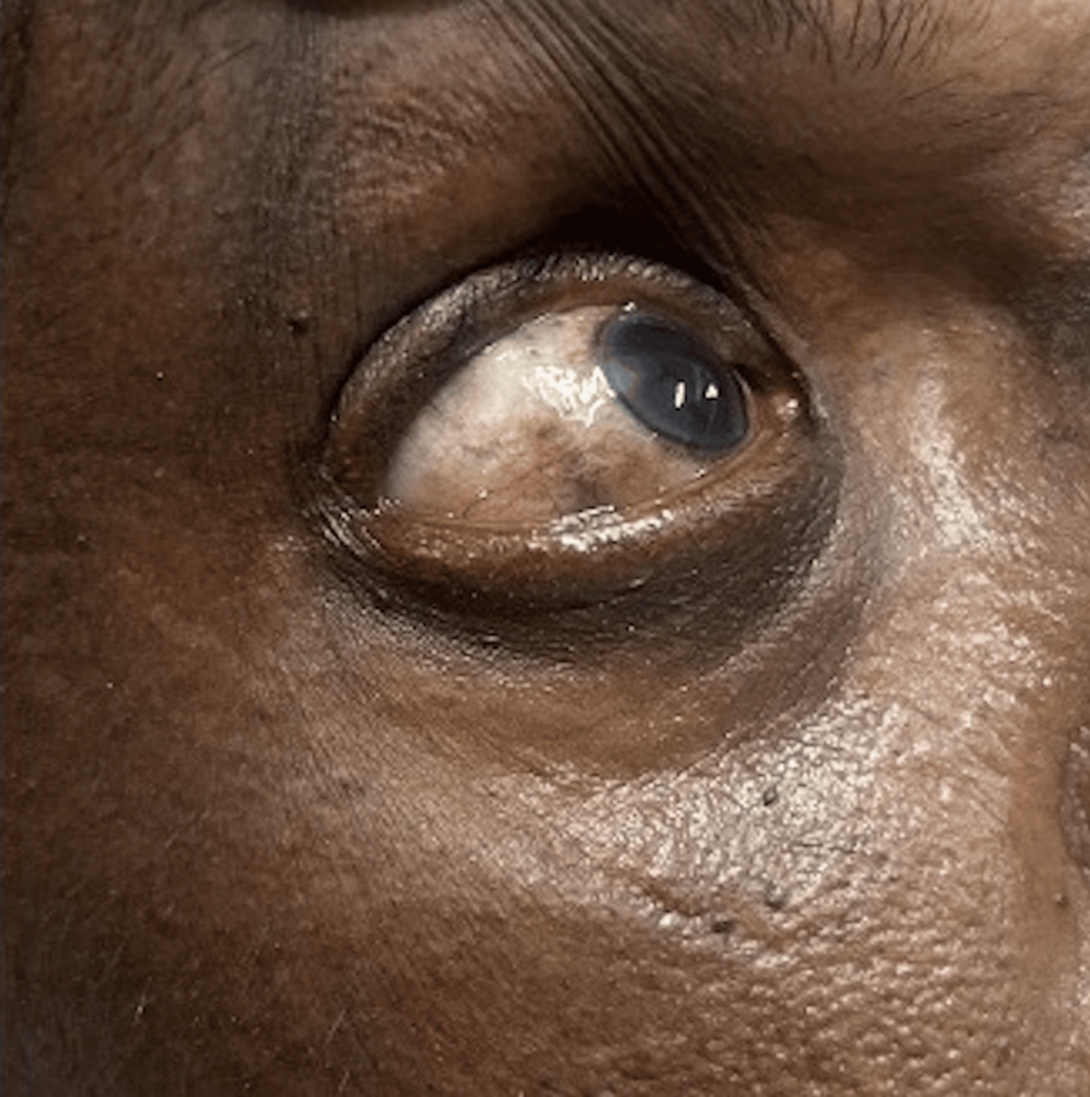
Blackish pigmentation of the sclera

On examination, his vitals were stable. The patient had blackish pigmentation of the sclera (Figure 1), and pallor was present. Abdominal examination revealed a tender and full bladder. Cardiovascular, respiratory and neurological examinations were unremarkable. Fundus examination revealed no evidence of diabetic retinopathy.

**Figure 2:**
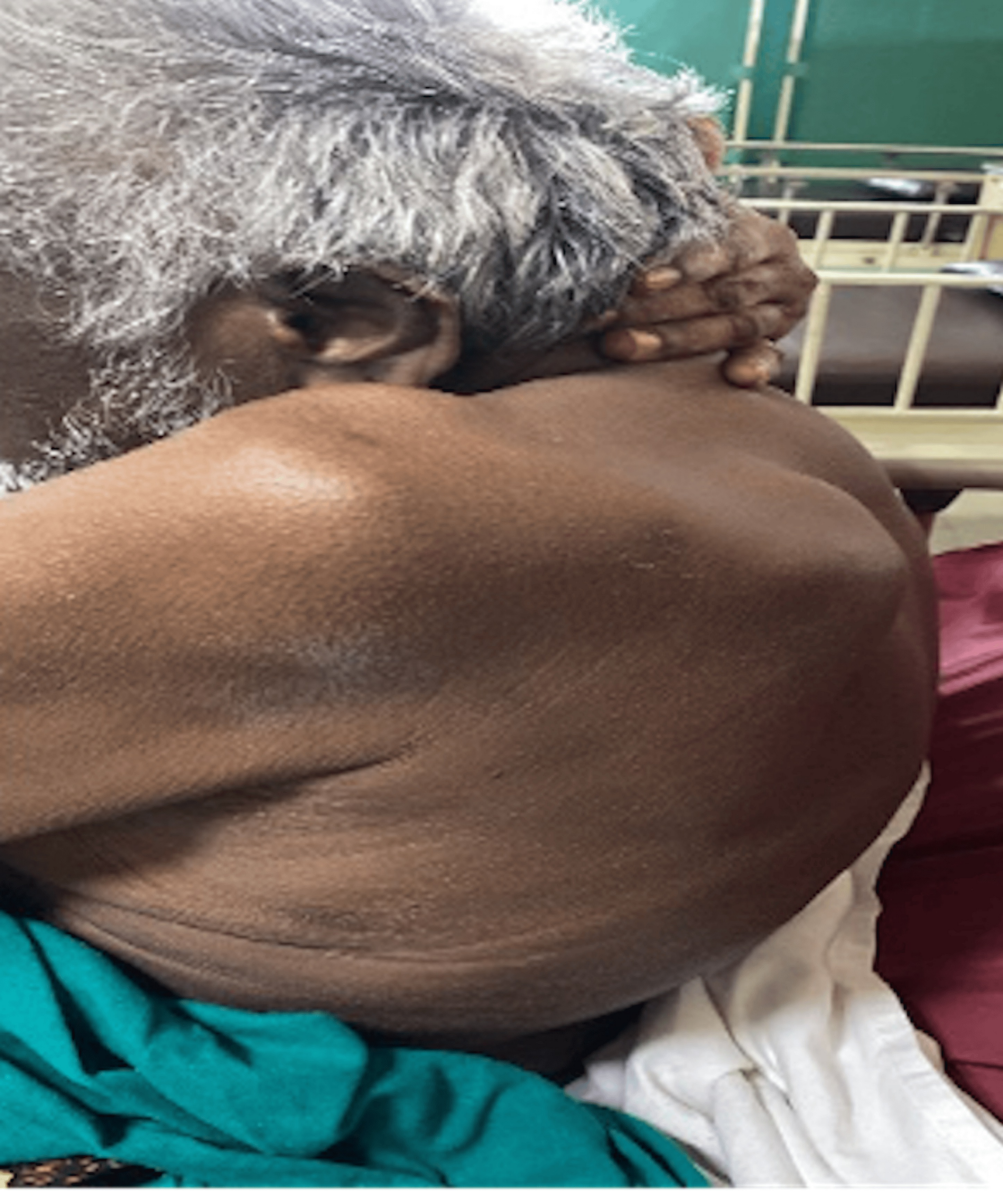
Kyphosis of the dorso-lumbar spine

**Figure 3:**
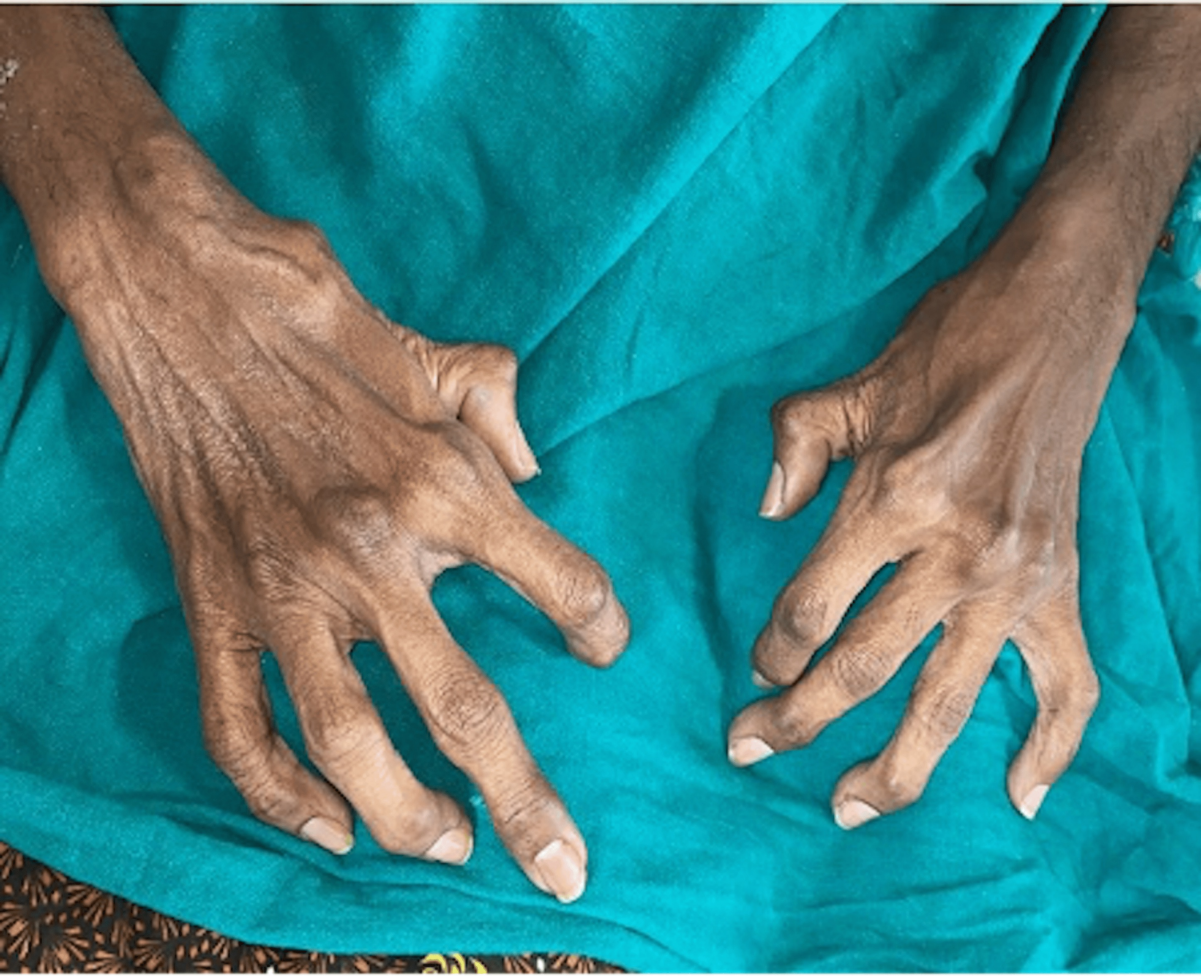
Small joint involvement of both hands

Musculoskeletal examination revealed kyphoscoliosis of the dorso-lumbar spine (Figure 2), flexion deformity of the interphalangeal joint of the thumb, deformity in the distal interphalangeal (DIP) joints of both hands and flexion deformity of the DIP joints and proximal interphalangeal (PIP) joints of other fingers of both hands (Figure 3). He also had a limited range of motion of the right glenohumeral joint, bilateral wrist joints, the dorso-lumbar spine and both knees.

Biochemical and metabolic panel revealed mild anaemia (haemoglobin 10.2 g/dL) with low ferritin and transferrin saturation, suggestive of iron deficiency anaemia. His renal function and his random blood sugar were normal. HbA1c was 6%. His urine was drained via suprapubic cystostomy, and the urine analysis revealed the presence of bacteria with plenty of pus cells, and nitrite was positive, suggestive of urinary tract infection. There were no casts in the urine. He had albuminuria as evidenced by urine dipstick analysis, and his urine protein creatinine ratio was 0.8. The rest of the labs were unremarkable.

CT scan of the abdomen showed multiple calculi within the prostatic urethra, two calculi within the bulbar urethra, which explained the acute urinary retention.

**Figure 4:**
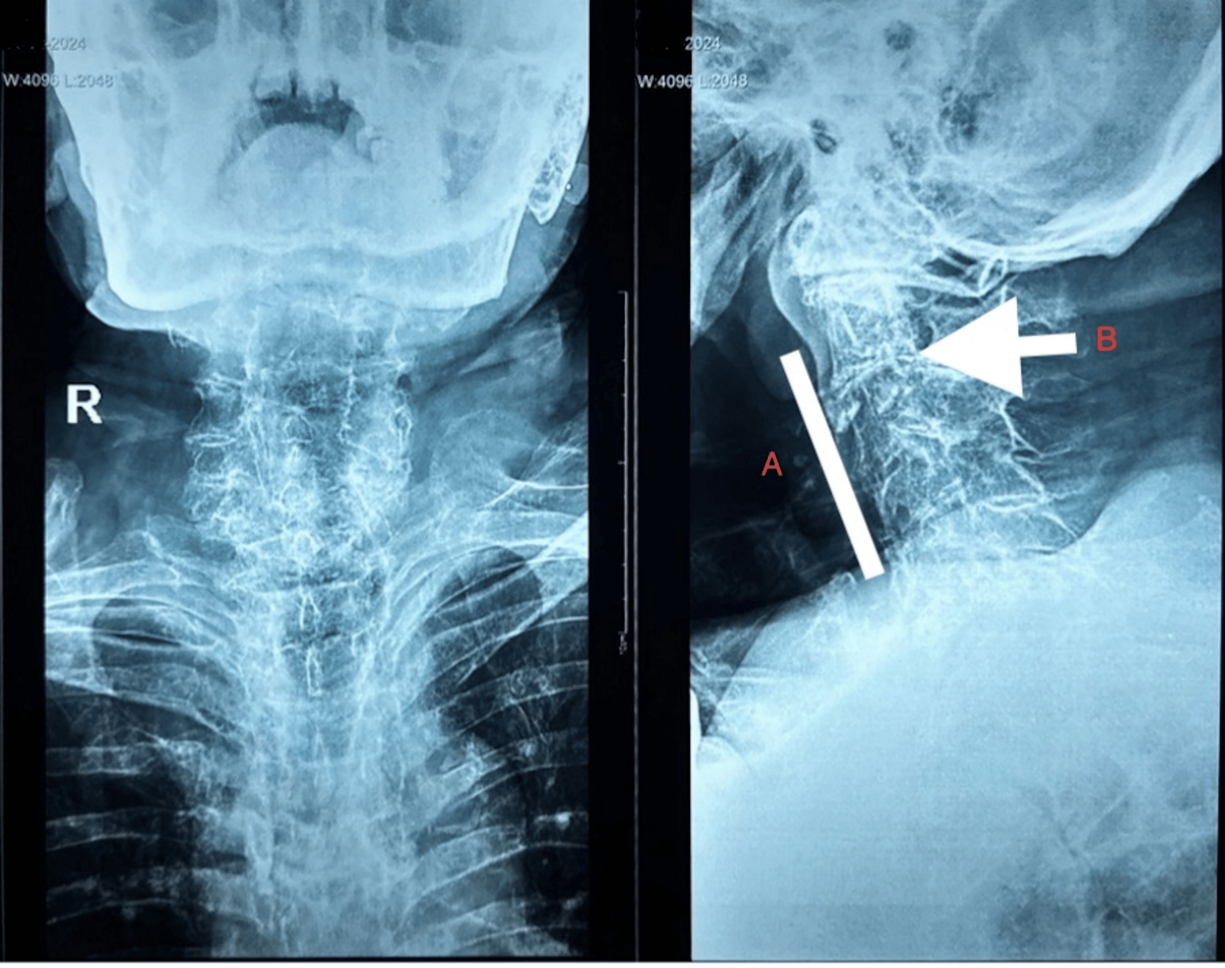
X-ray of the cervical spine showing A) straightening and B) narrowing of the intervertebral disc space

X-ray of the cervical spine showed narrowing of intervertebral spaces and straightening (Figure 4). 

**Figure 5:**
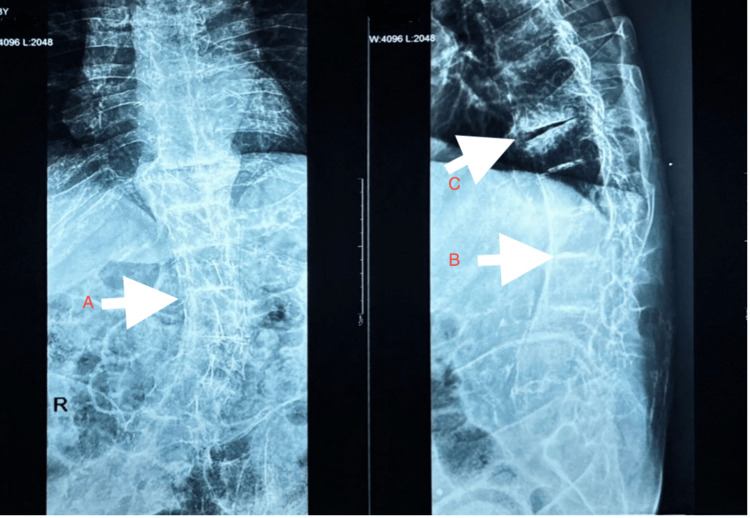
X-ray of the dorso-lumbar spine showing ankylosis, A) scoliosis, B) intervertebral disc calcification and C) subchondral sclerosis

X-ray of the dorso-lumbar spine (Figure 5) showed kyphoscoliosis, calcification of intervertebral discs, subchondral sclerosis and disc space narrowing.

**Figure 6:**
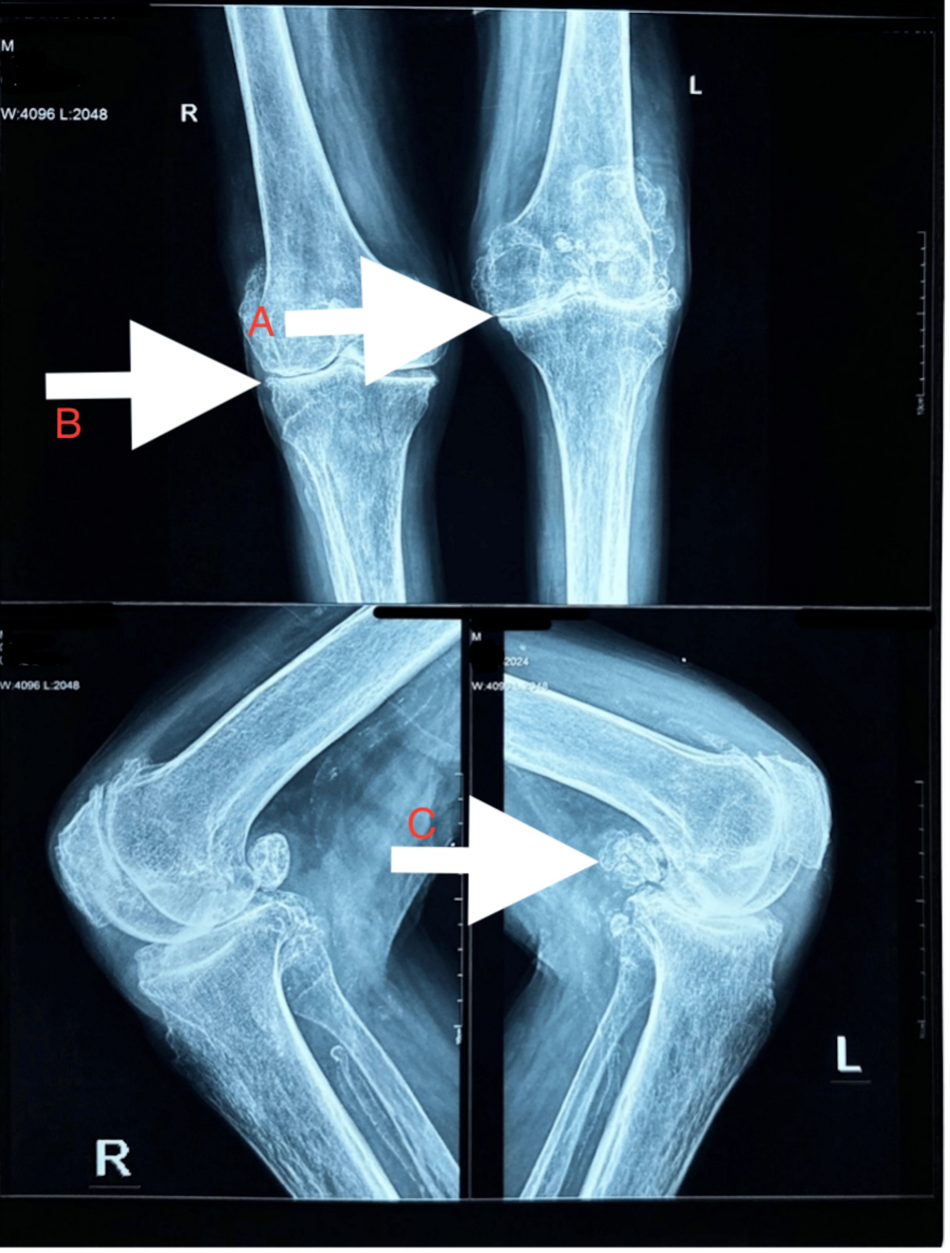
X-ray of bilateral knee (AP view above and lateral view below) showing A) joint space reduction, B) osteophyte and C) loose bodies

X-ray features often resemble those of ankylosing spondylitis, the difference being that the sacroiliac joint is spared. X-ray of the knee joint (Figure 6) showed space narrowing and multiple osteophytes.

**Figure 7:**
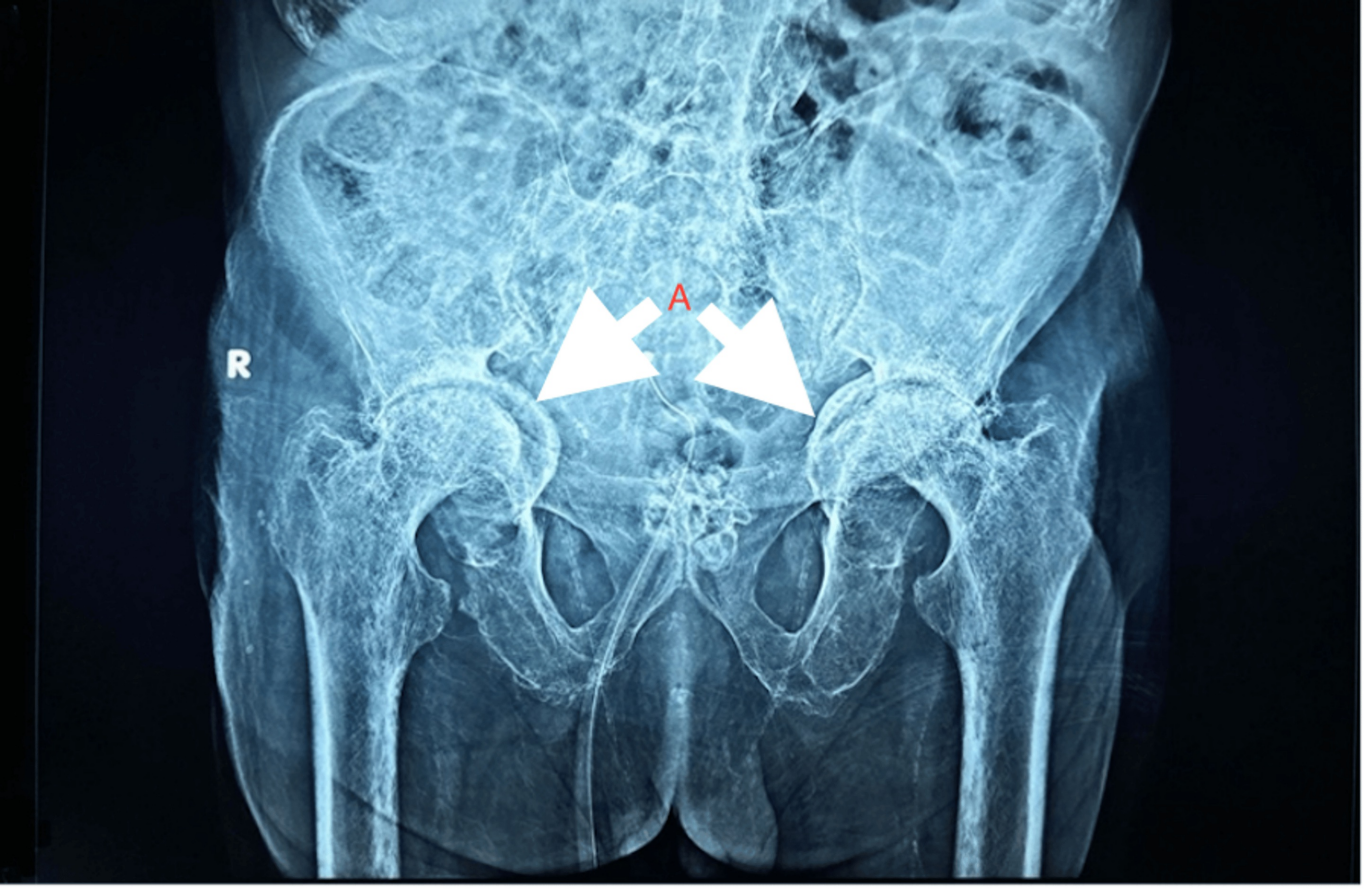
X-ray of hip with bilateral pelvis of patient showing resorption and flattening of the bilateral hip joint with A) protrusio acetabuli

**Figure 8:**
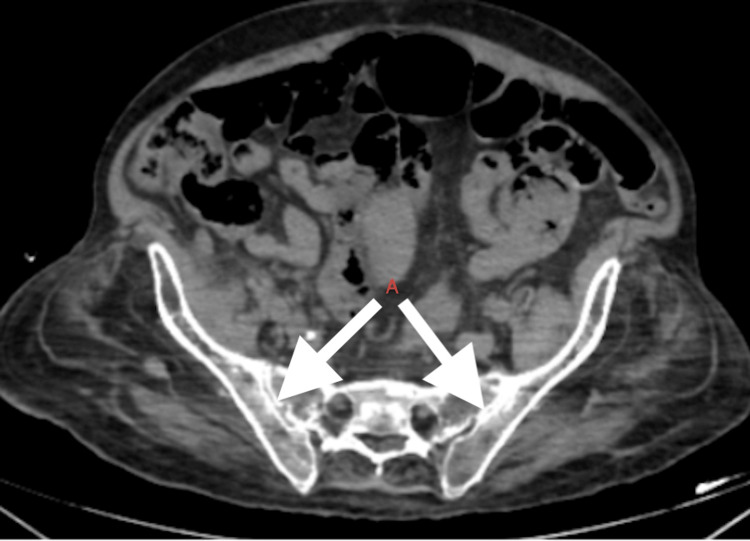
CT scan of bilateral A) sacroiliac joints

Additionally, the visualised portion of the vertebrae revealed dorso-lumbar scoliosis of the vertebra with convexity towards the left side, calcification of the anterior longitudinal ligament, multiple continuous flowing osteophytes with diffuse ankylosis in the visualised spine and osteoarthritis of hip joints (Figure 7). The sacroiliac joint did not show involvement (Figure 8).

**Figure 9:**
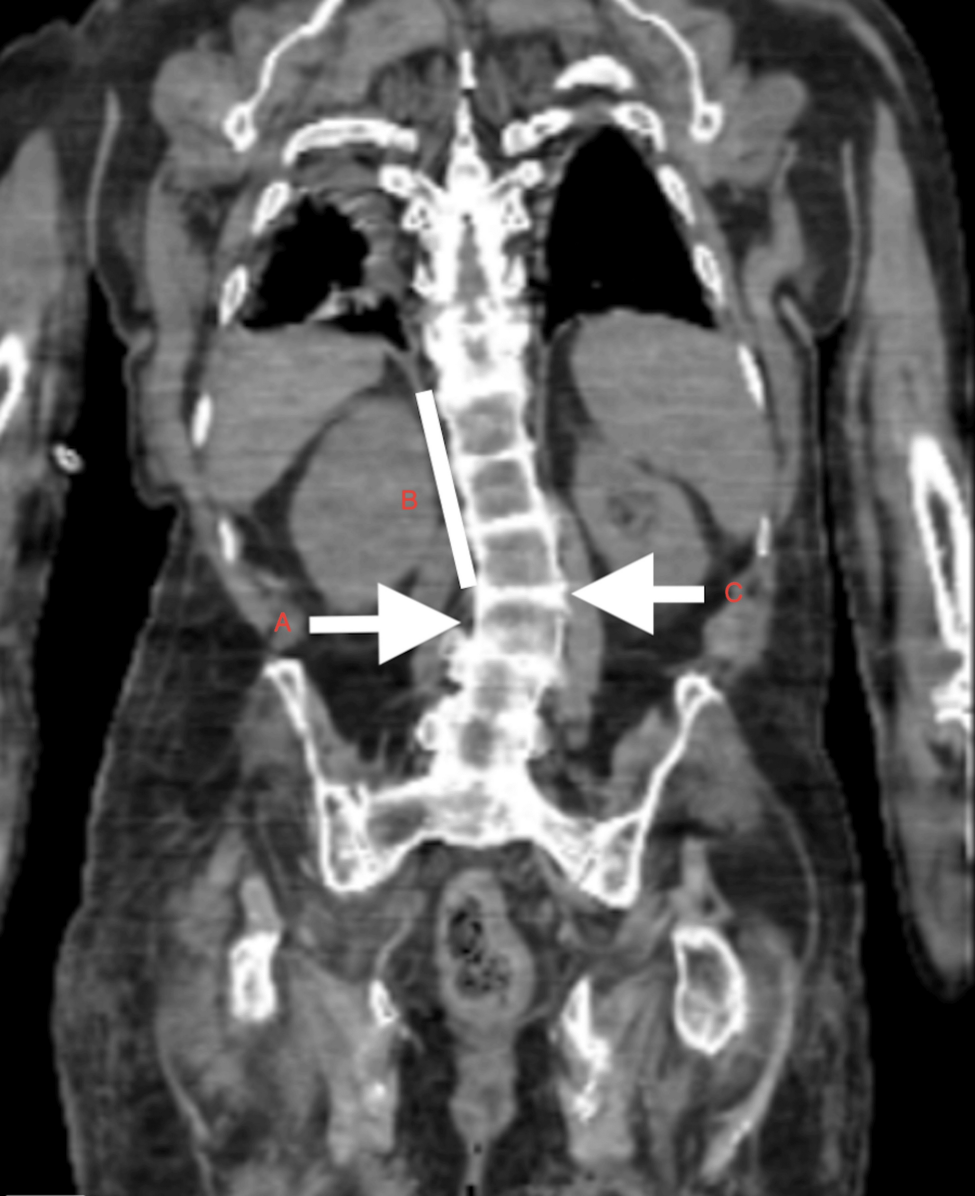
CT of dorso-lumbar spine showing A) dorso-lumbar scoliosis, B) ankylosis, diffuse osteopenia, disc space narrowing and C) multiple intervertebral disc space calcification and osteophytes

**Figure 10:**
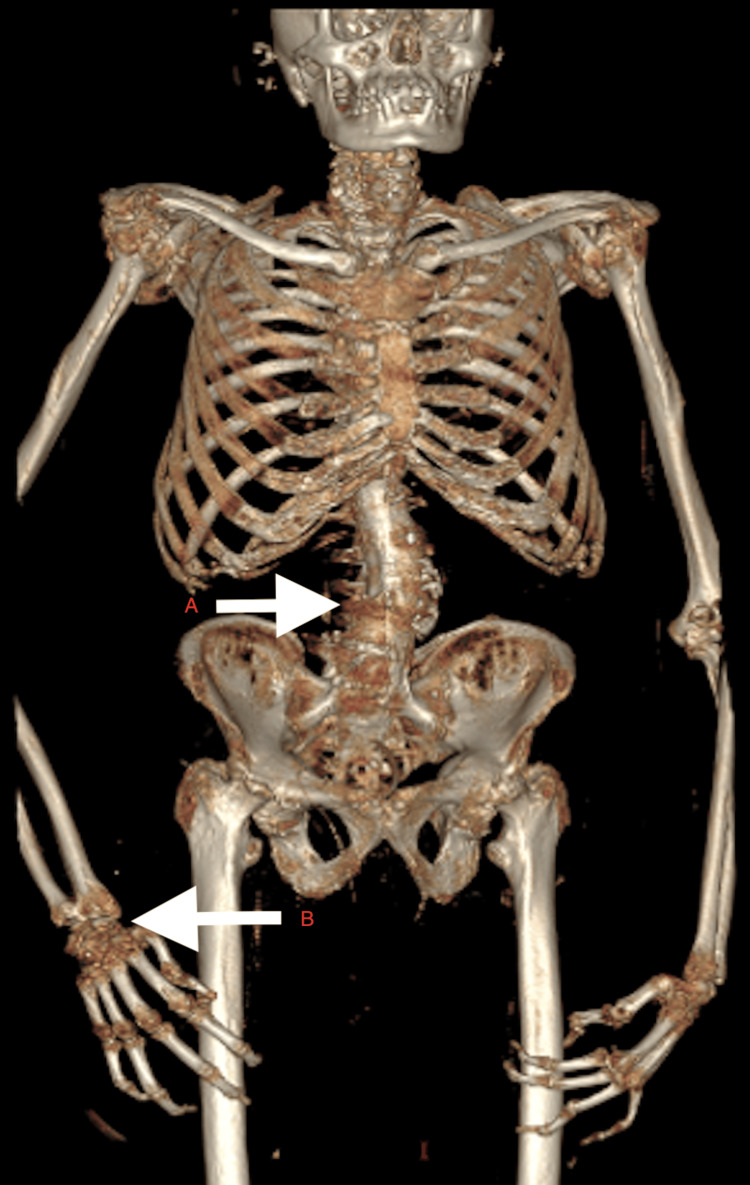
3D reconstruction AP view showing A) axial skeleton and B) appendicular skeleton involvement

**Figure 11:**
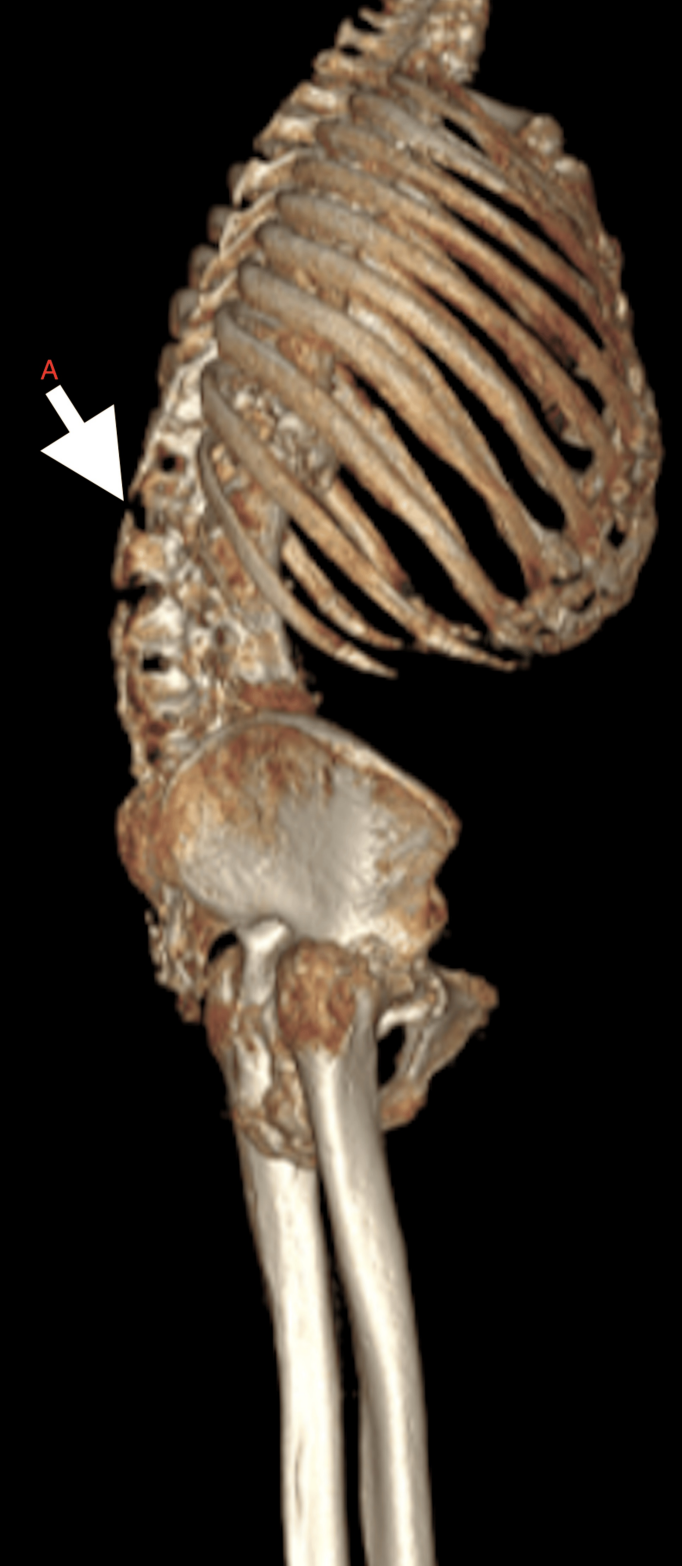
3D reconstruction lateral view showing A) kyphosis of the dorso-lumbar spine

CT scan of the dorso-lumbar spine showed dorso-lumbar scoliosis of the vertebra with convexity towards the left side, extensive ankylosis, degenerative changes in the form of diffuse osteopenia, disc space narrowing, multiple intervertebral disc space calcification and osteophytes involving entire vertebrae (Figure 9). 3D CT reconstruction of the skeletal system depicted the extensive involvement of the axial and appendicular skeleton, with the involvement of small and large joints equally (Figures 10-11).

Urine HGA assay was found to be elevated (1161 mmol/L, normal < 0.99). The diagnosis of alkaptonuria was confirmed based on the elevated urinary homogentisate level and the radiological findings.

**Figure 12:**
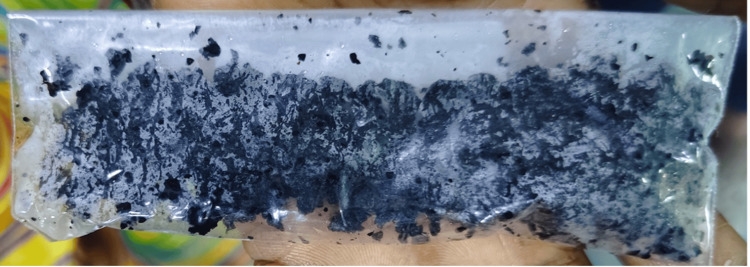
Post-percutaneous cystolithotripsy (PCCL) specimen, showing alkapton bodies (blackish stones)

The patient later underwent percutaneous cystolithotripsy. Following the procedure, irregular, hard, blackish stones made of calcium phosphate were retrieved, suggestive of alkapton bodies (Figure 12). Given the advanced stage of musculoskeletal manifestations, the prognosis was poor. Hence, the patient was started on physiotherapy along with analgesics to help with mobility.

## Discussion

Alkaptonuria results in ochronosis, homogentisic aciduria and ochronotic arthropathy. These features manifest at different ages, with the earliest being the presence of HGA in urine. Musculoskeletal manifestations are delayed until the fourth decade primarily because of the efficient renal tubules that excrete several grams of HGA per day until the fourth decade [5]. Osteopenia, osteoporosis, tendinopathy and arthropathy are the three main musculoskeletal manifestations of alkaptonuria. Musculoskeletal symptoms begin with axial skeleton involvement, followed by appendicular skeletal involvement, tendinopathy, and eventually lead to osteopenia and osteoporosis, although the sequence may vary [6].

Our patient presented in his 60s with a history of urine turning black on standing, chronic back ache and progressive stiffness, which was undiagnosed and untreated till presentation. This led to irreversible degenerative changes like kyphoscoliosis, subchondral sclerosis and ankylosis, restricting his mobility.

Arthropathy

Arthropathy due to ochronosis manifests as early-onset appendicular joint or axial joint pain, associated with decreased range of motion, swelling and morning stiffness. Ochronotic pigment gets deposited in the joints, in the articular and periarticular cartilage, ligaments that are collagen-rich and the menisci. Oxidised HGA deposition occurs preferentially in the senescent cartilage with decreased metabolism. This makes the cartilage matrix further susceptible to pigmentation in response to tissue injury, including microtrauma [7]. The cartilage and the menisci lose elasticity and are unable to withstand mechanical strain. Hence, the cartilage gets disintegrated and forms loose bodies. These fragments get lodged in the synovial membrane. This leads to fibrosis or chondromatosis forming osteophytes or subchondral cysts [8].

Several theories have been proposed regarding the pathophysiology of ochronotic arthropathy. One theory postulated that HGA oxidation produces free radicals, causing inflammatory damage to the tissues and that ochronosis affects tissue turnover in the cartilage, and this in turn accelerates the disease initiation and progression. Taylor et al. [7] showed that there are low levels of extractable matrix protein in cartilages and a very low turnover state in cartilages of patients with alkaptonuria when compared to patients with osteoarthritis. Shimizu et al. [9] reported the disintegration of proteoglycan within the cartilage matrix in ochronotic arthropathy. They proposed that joint degeneration in this disease occurs as a result of clefts in the central cartilage matrix, making it more susceptible to collapse. The resultant degeneration of intervertebral discs leads to a reduction in the intervertebral space and subsequent development of bony ankylosis connecting adjacent vertebrae. Another theory proposes that the ochronotic pigment acts like an irritant, causing inflammation, which leads to joint destruction.

Ochronotic arthropathy typically manifests first in the lumbar spine in the form of morning stiffness and pain. As the disease progresses, there is a gradual loss of lumbar lordosis and an increase in thoracic kyphosis (Figure 2). Degenerative changes in the spine, particularly osteophyte formation along the dorsal aspect of the vertebral body and uncinate process, may compromise the neural canal, subsequently causing myelopathy. Though uncommon, some reports have documented lumbar disc prolapse. 

Key radiologic features include intervertebral disc calcification at multiple levels as seen in our patient (Figure 5), vacuum phenomena characterised by radiolucent gas collections indicative of severe degeneration and progressive narrowing of the intervertebral spaces. In chronic patients, apophyseal joint degeneration, disc space reduction, kyphosis and bony ankylosis can develop, mimicking ankylosing spondylitis as evidenced in our patient (Figure 5). However, it differs in that it generally spares the sacroiliac joints (Figure 8) [10] and lacks features such as bamboo spine, annular ossification and syndesmophytes. In advanced cases, this degeneration can lead to vertebral fusion, resulting in a height loss of up to 15 cm. 

Among the peripheral joints affected in alkaptonuria, the knee is the most commonly affected. However, knee involvement usually occurs years after spinal symptoms begin. Similar to axial joint patients, these patients present with pain, stiffness and decreased mobility. It is due to the progressive degeneration and eventual collapse of both the lateral and medial compartments. In about half of the cases, synovial effusion is present, and aspiration of synovial fluid may reveal floating black pigment particles, a phenomenon known as the ground pepper sign [11]. The affected cartilage takes on a distinctive black discolouration and becomes brittle. Radiographic imaging of the knee shows joint space narrowing (Figure 6) accompanied by loose osteochondral bodies. Retropatellar joint space narrowing due to femoral cartilage degeneration is another characteristic feature seen in ochronotic arthropathy of the knee.

Tendinopathy

Tendons are commonly involved in alkaptonuria due to high collagen concentration. The Achilles tendon and the patella are the two most common sites. The accumulation of HGA in type 1 collagen hinders cross-linking of collagen, which weakens the structural integrity of collagen, leading to tendon rupture as observed by Manoj Kumar and Rajasekaran et al [12]. Medical treatment to prevent tendinopathy is limited, similar to arthropathy, and surgical treatment involves repair of ruptured ligaments and tendons.

Osteopenia and osteoporosis

Alkaptonuria leads to decreased bone mineral density (BMD), which leads to fragility fractures. Aliberti et al. [13] showed that there is an imbalance in the metabolism of bone, as evidenced by elevated urinary NTx, suggestive of resorption of bone. There was no evidence of a decrease/increase in osteoblastic activity. This imbalance subsequently leads to osteopenia and osteoporosis. They hypothesised that accelerated bone loss may be due to the HGA polymer deposit in the bone matrix and osteocytes [13]. Periodic evaluation of femoral BMD and bone resorption biomarkers in affected persons from a young age should be considered to plan a preventive therapy. 

Diagnosis of alkaptonuria is delayed as the musculoskeletal manifestations of the disease mimic other types of arthritis, as was the case in our patient, who was treated as a case of rheumatoid arthritis for years before being diagnosed with alkaptonuria. At present, treatment options to prevent complications of alkaptonuria are limited. Antioxidants, vitamin C and nitisinone are some of the suggested pharmacotherapies that offer some benefit to the patient. HGA oxidation may be prevented by vitamin C and other antioxidants, as suggested by Morava et al. [14]. A low phenylalanine and tyrosine diet was also advocated by them. However, the chronic effects of such a diet on the outcome of alkaptonuria have not been proven conclusively.

**Figure 13:**
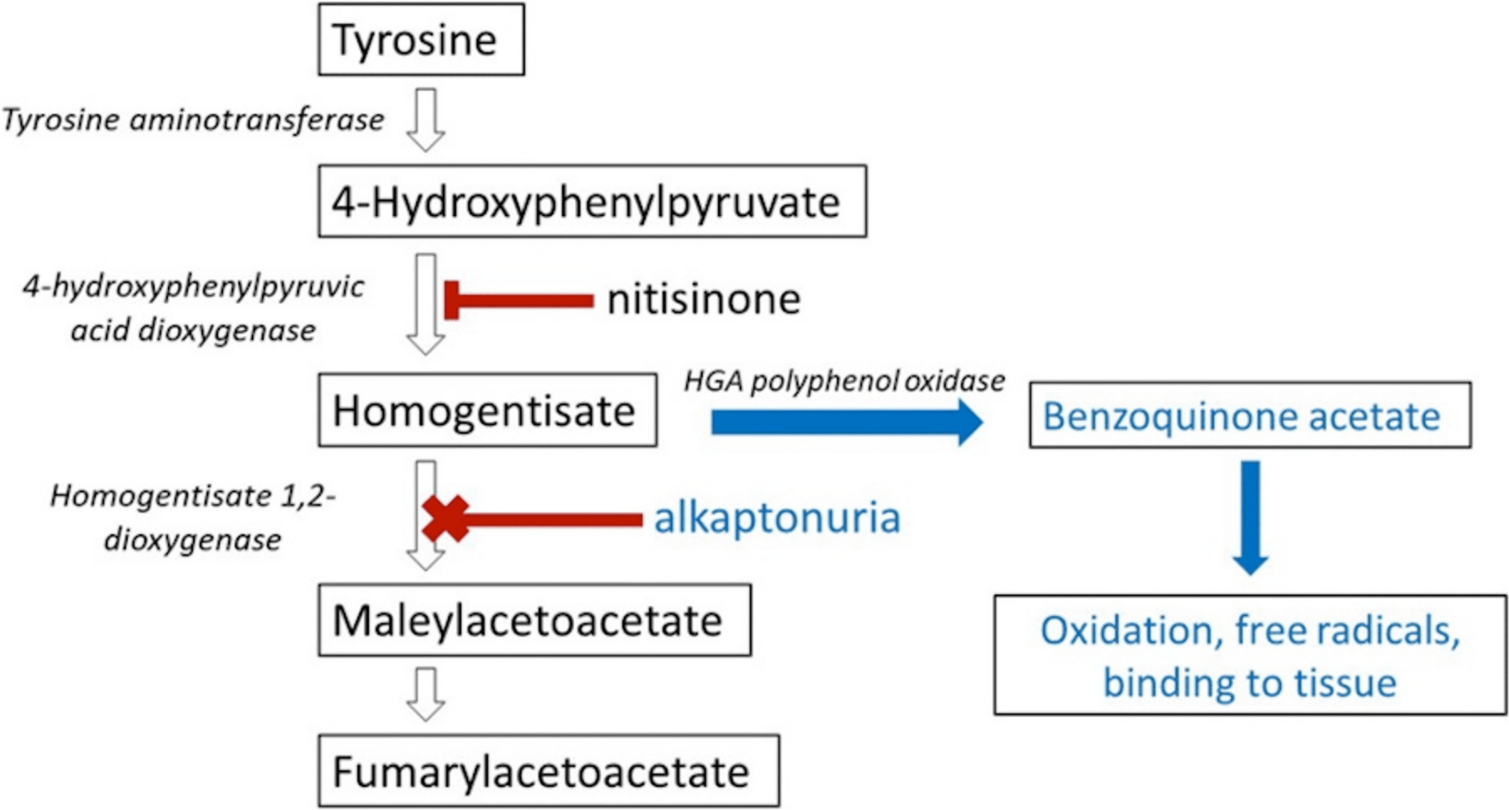
Tyrosine degradation pathway Reference: [1]

4-Hydroxyphenylpyruvate dioxygenase converts 4-hydroxyphenylpyruvate to HGA [1] (Figure 13), and it is competitively inhibited by nitisinone. It is currently used to treat type 1 tyrosinemia. Oral administration of nitisinone reduces urinary HGA secretion, and Keenan et al. [15] also found that nitisinone treatment arrests disease progression if treatment is started from midlife in adult mice, but it does not reverse the disease process. However, they found that nitisinone treatment initiated since birth can prevent ochronosis in adult mice [15]. 

Increased tyrosine levels due to nitisinone treatment are toxic to the skin, nervous system, cornea and conjunctiva. Hence, a low phenylalanine and tyrosine diet should be followed when taking nitisinone.

## Conclusions

Alkaptonuria, though a rare metabolic disorder, can lead to significant musculoskeletal complications when left undiagnosed and untreated, causing significant morbidity. It predominantly affects weight-bearing joints and the spine. Cardinal musculoskeletal manifestations of ochronotic arthropathy include chronic back pain, stiffness, joint degeneration, and early-onset osteoarthritis. This case highlights the importance of recognising musculoskeletal manifestations of alkaptonuria, enabling early diagnosis and targeted management to preserve joint function and slow the progression, which improves the quality of life in affected patients. The treatments currently in use are vitamin C, antioxidants, nitisinone and surgical management.
